# Intraspecies Transmission of BASE Induces Clinical Dullness and Amyotrophic Changes

**DOI:** 10.1371/journal.ppat.1000075

**Published:** 2008-05-23

**Authors:** Guerino Lombardi, Cristina Casalone, Antonio D' Angelo, Daniela Gelmetti, Gloria Torcoli, Ilaria Barbieri, Cristiano Corona, Elisa Fasoli, Alessia Farinazzo, Michele Fiorini, Matteo Gelati, Barbara Iulini, Fabrizio Tagliavini, Sergio Ferrari, Maria Caramelli, Salvatore Monaco, Lorenzo Capucci, Gianluigi Zanusso

**Affiliations:** 1 Istituto Zooprofilattico Sperimentale della Lombardia e dell' Emilia Romagna, Brescia, Italy; 2 Centro di Referenza Nazionale per le Encefalopatie Animali, Istituto Zooprofilattico Sperimentale del Piemonte, Liguria e Valle D' Aosta, Turin, Italy; 3 Dipartimento di Patologia Animale, Università degli Studi di Torino, Grugliasco, Italy; 4 Department of Neurological and Visual Sciences, Section of Clinical Neurology, University of Verona, Verona, Italy; 5 Fondazione IRCCS Istituto Nazionale Neurologico “Carlo Besta”, Milan, Italy; University of Alberta, Canada

## Abstract

The disease phenotype of bovine spongiform encephalopathy (BSE) and the molecular/ biological properties of its prion strain, including the host range and the characteristics of BSE-related disorders, have been extensively studied since its discovery in 1986. In recent years, systematic testing of the brains of cattle coming to slaughter resulted in the identification of at least two atypical forms of BSE. These emerging disorders are characterized by novel conformers of the bovine pathological prion protein (PrP^TSE^), named high-type (BSE-H) and low-type (BSE-L). We recently reported two Italian atypical cases with a PrP^TSE^ type identical to BSE-L, pathologically characterized by PrP amyloid plaques and known as bovine amyloidotic spongiform encephalopathy (BASE). Several lines of evidence suggest that BASE is highly virulent and easily transmissible to a wide host range. Experimental transmission to transgenic mice overexpressing bovine PrP (Tgbov XV) suggested that BASE is caused by a prion strain distinct from the BSE isolate. In the present study, we experimentally infected Friesian and Alpine brown cattle with Italian BSE and BASE isolates via the intracerebral route. BASE-infected cattle developed amyotrophic changes accompanied by mental dullness. The molecular and neuropathological profiles, including PrP deposition pattern, closely matched those observed in the original cases. This study provides clear evidence of BASE as a distinct prion isolate and discloses a novel disease phenotype in cattle.

## Author Summary

For approximately two decades, bovine spongiform encephalopathy (BSE), now termed classical BSE (BSE-C), has been regarded as the only and exclusive prion disorder affecting cattle. However, over the last 4 years, two additional bovine prion strains, bovine amyloidotic spongiform encephalopathy (BASE, also named BSE-L) and BSE-H, have been discovered and characterized in Canada, the United States, Japan, and nine European countries, which applied an active surveillance program on slaughtered cattle. Although a total of 20 BSE-L and 16 BSE-H have been reported to date, the disease phenotype of these conditions remains largely unknown. Intriguingly, recent evidence has been provided that the BSE-C and BASE strains disclose converging properties after transmission to inbred mice. Here, we show that intraspecies transmission of BASE induces a disease phenotype characterized by dullness and progressive amyotrophy, the latter highly suggestive of a motor neuron disorder. This is at variance with the over-reactivity and hypersensitivity, but not muscle changes, observed in BSE-transmitted cattle. This study confirms that BASE and BSE represent two distinct prion disorders in cattle with diverging molecular features and disease phenotypes.

## Introduction

Prion diseases, or transmissible spongiform encephalopathies (TSEs), are mammalian neurodegenerative disorders of sporadic, genetic, or infectious origin characterized by accumulation and deposition of an abnormal isoform (PrP^TSE^) of the cellular prion protein (PrP^C^) in the brain [1]. TSEs include a wide range of animal and human disorders, such as BSE in cattle, scrapie in sheep and goats, chronic wasting disease in deer and elk, and Creutzfeldt-Jakob disease (CJD) in humans [1].

First identified in 1986 in the UK, BSE has been confirmed in over 180,000 cases, although more than one million cattle have been estimated to be infected [2]. Evidence of the spread of the BSE agent across certain mammalian species, including humans, indicates that this disease is a major animal and human public health issue [3]–[5]. Common neurological signs in cattle include apprehension, hyperaesthesia, kicking, and pelvic limb ataxia, accompanied by general signs such as reduced milk yield and loss of conditions. In all cases, progression to behavioural, sensory and posture/movement alterations led to death within a few months [6],[7].

Early transmission studies showed that isolates from field BSE cases and variant CJD (vCJD), its human counterpart, were all caused by a single prion strain [4]. In addition, PrP^TSE^ from BSE and vCJD cases exhibited a distinctive glycotype signature, with high glycosylation site occupancy and similar electrophoretic mobility of the unglycosylated protease-resistant PrP^TSE^ fragment [8]. These PrP^TSE^ traits have been used as biochemical indicators of the BSE prion strain.

Until recently, monitoring of BSE in cattle was accomplished by passive surveillance and pathological confirmation of suspected clinical cases. In 2001, the European Community imposed an active surveillance system based on biochemical tests of brain tissues from all slaughtered cattle over 30 months of age. This strategy led to the recent identification of new PrP^TSE^ types, provisionally termed as “type-H” and “type-L” according to the electrophoretic migration of the unglycosylated proteinase K-resistant PrP^TSE^, which is higher (BSE-H) or lower (BSE-L) than classical BSE (BSE-C) [9]–[11]. An additional distinctive signature of type-H and type-L is the even representation of di-, mono-, and unglycosylated PrP^TSE^ species.

In 2004, we described two aged asymptomatic Italian cattle of Piemontese and Alpine brown breeds neuropathologically characterized by the presence of PrP-amyloid plaques [12]. This new pathological phenotype, named BASE, was characterized by marked involvement of olfactory areas, hippocampus, and thalamus, with relative sparing of the brainstem. The molecular signature of BASE PrP^TSE^ was similar to that later detected in BSE-L cases [11]. A feature shared between BASE, a condition both well-defined molecularly and pathologically, and “L-type” cases, defined only on a molecular basis, is the older age of the affected animals (approximately 12 years) as compared to BSE cases (5–6 years).

Recent studies have shown that BASE and “L-type” isolates exhibit similar biological properties upon transmission to Tgbov XV, and have shorter incubation period and survival time than BSE; these findings are suggestive of a single prion strain for BASE and BSE-L [10],[13]. In contrast, the H-type phenotype showed an unusually long incubation period in Tgbov XV [10].

To date, all of the available demographic and molecular evidence strongly suggests that H-type BSE and BASE-L represent sporadic forms of bovine spongiform encephalopathies [14].

Human susceptibility to BASE has been suggested by experimental transmission to primates and to PrP humanized transgenic mice [14].

Here we inoculated cattle of different breeds with brain homogenates from Italian BASE and BSE cases, in order to assess the strain attributes and disease phenotype of the above isolates in their natural hosts.

## Results

### Genetic analysis

All Friesian cattle were homozygous for six octapeptide repeat copies, and three cattle carried a silent mutation at codon 78 (CAG/CAA). Four out of six Alpine brown cattle were homozygous for six octapeptide repeat copies; one animal carried 6/7 and another 5/7 octapeptide repeat copies. Four different silent mutations were found at codons 78 (CAG/CAA), 23 (CTC/CTT), 95 (CCA/CCC), and 77 (GGT/GGC) in four cattle. Homozygosity for 23 bp and 12 bp deletion alleles was present in three Friesian cattle. Results of these genetic studies are summarized in Table 1.

**Table 1:** Genetics of cattle.

Code	Breed	Inoculum	Octarepeats	Variation	Codon	*12-indel*	*23-indel*
258	Friesian	BSE	6/6	CAG/CAA	78 Q/Q	214:214	190:190
326	Friesian	BSE	6/6	-	Wt	202:202	167:167
329	Friesian	BSE	6/6	CAG/CAA	78 Q/Q	202:214	167:190
254	Friesian	BASE	6/6	CAG/CAA	78 Q/Q	214:214	190:190
259	Friesian	BASE	6/6	-	Wt	202:202	167:167
261	Friesian	BASE	6/6	-	Wt	202:202	167:167
330	Friesian	Saline	6/6	-	Wt	202:214	167:190
388	Friesian	Saline	6/6	-	Wt	202:214	167:190
340	Alpine brown	BSE	6/6	CTC/CTT CAG/CAA	23 L/L 78 Q/Q	214:214	167:190
817	Alpine brown	BSE	5/7	GGT/GGC CCA/CCC	77 G/G 95 P/P	214:214	167:190
844	Alpine brown	BSE	6/7	CCA/CCC	95 P/P	214:214	190:190
816	Alpine brown	BASE	6/6	-	Wt	202:214	167:190
994	Alpine brown	BASE	6/6	CAG/CAA	78 Q/Q	202:214	167:190
995	Alpine brown	BASE	6/6	-	Wt	214:214	190:190

### Incubation periods and clinical disease duration

A total of twelve cattle, two groups of three Alpine brown and three Friesian, intracerebrally inoculated with either BSE or BASE, developed neurological signs and were killed at the terminal stage of disease (Table 2). In contrast, two saline inoculated Friesian cattle are free of clinical signs at the time of writing, i.e. 42 months post-inoculation. In BASE-treated cattle the clinical disease duration was shorter than in BSE-inoculated animals; however, caution in evaluating these differences is dictated by the low number of experimental animals in addition to the undetermined infectivity titre of the inocula.

**Table 2:** Clinical phenotype, incubation period and clinical duration in BSE- and BASE-infected cattle.

Inoculum/Attack rate	Code/Breed*	Clinical signs at onset	Progression of clinical signs	Incubation period (days)	Clinical duration (days)
BSE 100%	258-F	Nervousness, hypersensitivity	Apprehension, hypersensitivity to tactile, acoustic and visual stimuli	524	180
	326-F			660	45
	329-F			640	150
				Mean: 608	Mean: 125
	340-Ab	Nervousness, hypersensitivity	Apprehension, hypersensitivity to tactile, acoustic and visual stimuli	503	120
	817-Ab			514	180
	844-Ab			493	60
				Mean: 503	Mean: 120
BASE 100%	254-F	Dullness	Downer cattle	461	10
	259-F	Dullness, amyotrophy	Dullness, amyotrophy, hypersensitivity to facial tactile stimuli	480	60
	261-F			470	30
				Mean: 470	Mean: 33
	816-Ab	Dullness, amyotrophy	Dullness, amyotrophy, hypersensitivity to facial tactile stimuli	551	30
	994-Ab			530	60
	995-Ab			525	105
				Mean: 535	Mean: 65

* F denotes Friesian; Ab denotes Alpine brown.

### Clinical features

**Figure 1:**
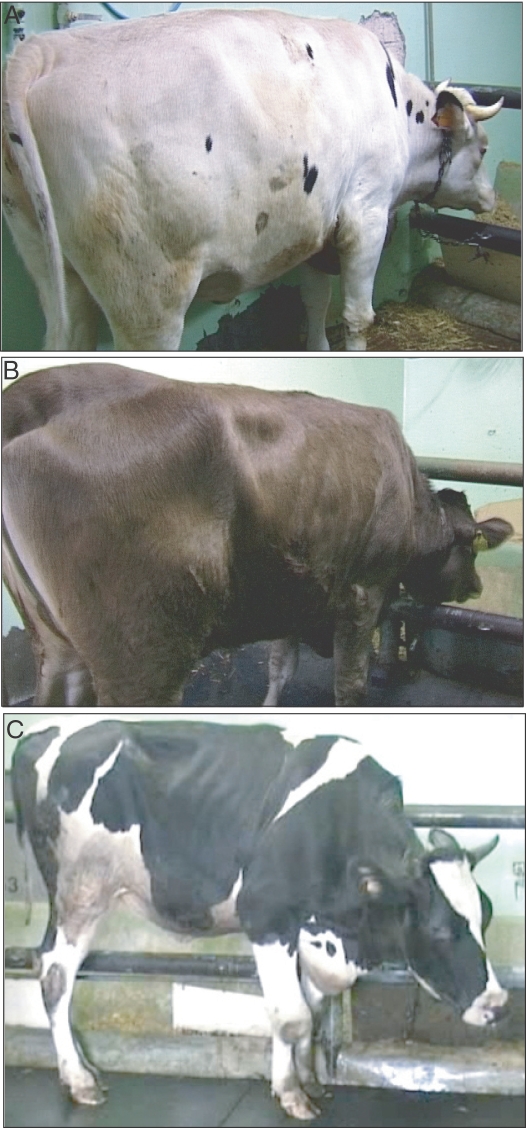
BSE and BASE infected cattle. (A) BSE Friesian (#258) at 670 d.p.i., (B) BASE Alpine brown (#995) 600 d.p.i., and (C) BASE Friesian (#261) 585 d.p.i. BASE-infected cattle show muscle atrophy particularly severe at the hind limbs and pelvic girdle (B,C), while in BSE-challenged cattle (A) musculature is apparently normal.

In BSE-inoculated cattle, clinical signs at onset consisted of behavioural changes and hypersensitivity. As the disease progressed, major clinical signs included aggressiveness, frequent bellowing and head shaking, postural abnormalities, exaggerated blink reflex, generalized cutaneous hyperaesthesia, and stimulus-induced myoclonic jerks (Table 2 and Figure 1A and Video S1).

Conversely, early neurological signs in both Friesian and Alpine brown cattle inoculated with BASE consisted of fasciculations of gluteal muscles, a dull coat and postural and behavioural signs of depression, including low head carriage, mild kyphosis, and decreased alertness. With progression, muscle atrophy, beginning in the gluteal region and progressing to the paravertebral region and to other hind limb musculature became apparent (Figure 1B and 1C). Fore-limb muscles were relatively spared (Video S2). With the exception of the “downer” cattle, neither gait ataxia nor difficulties in rising were observed throughout the disease course.

Cattle showed an exaggerated response to facial touch or pinch, but not to light and sound stimuli. Observations via night filming showed that BASE cattle were prone to sudden falls. One Friesian cow (code # 254) showed a “downer” syndrome at onset.

### Biochemical characterization and regional distribution of PrP^TSE^

**Figure 2:**
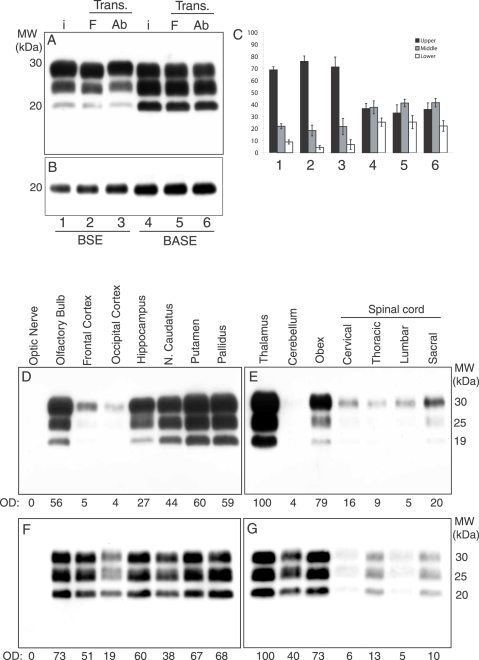
Biochemical analysis and regional distribution of PrP^TSE^ in BSE and BASE experimentally infected cattle. (A) Immunoblot with 6H4 of proteinase K-treated brain homogenates from BSE and BASE inocula (abbreviated as i) (lanes 1 and 4) from Friesian (abbreviated as F) and Alpine brown (abbreviated as Ab) inoculated with BSE (lanes 2 and 3, respectively), and BASE (5 and 6, respectively); (B) samples after PNGase F-treatment. (C) Relative proportions of the three PrP^TSE^ glycoforms in BSE and BASE inocula and in infected cattle. (Standard error bars correspond to variations of PrP^TSE^ glycoform in the inocula, as determined in six replicates. On the contrary, in Friesians and in Alpine browns cattle inoculated with BSE and BASE determination was performed on at least six different brain areas from each animal) (D–G) Brain regional distribution of PrP^TSE^ in BSE (D and E) and BASE (F and G) infected cattle. Values below each lane correspond to the relative percentage of PrP^TSE^ normalized against thalamus. MW, molecular weights; kDa: kilodaltons; OD: optical density.

Immunoblot analysis of proteinase K-treated (PK) brain homogenates from each BSE- and BASE-infected cattle revealed the presence of PrP^TSE^ in all sampled areas. However, all BSE-challenged animals showed a di-glycosylated-dominant PrP^TSE^ type, whereas in all BASE-inoculated cattle a mono-glycosylated-dominant PrP^TSE^ type was detected. In addition, the molecular mass of the PK-resistant unglycosylated fragment was identical to that of the original inoculum in each animal (Figure 2A–2C). In cattle infected with BSE, the highest amounts of PrP^TSE^ were observed in the thalamus, basal ganglia, obex, olfactory areas and hippocampus, whereas very low amounts were seen in cerebral cortices and cerebellum (Figure 2D and 2E). Differently from BSE, in BASE-infected cattle consistently high amounts of PrP^TSE^ were observed in cerebral cortex, hippocampus, and cerebellum (Figure 2F and 2G). In both groups, low amounts of PrP^TSE^ were found in the spinal cord.

In all experimentally infected animals, no PrP^TSE^ was detected in peripheral tissues, including cervical and mesenteric lymph nodes, spleen, thymus, liver, lung, peripheral nerves and forelimb and hind limb muscles, either by standard Western blot analysis or following phosphotungstic acid precipitation.

### Pathological and immunohistochemical findings

Typical neuropathological changes, including spongiosis and gliosis were detected in all cattle (Figure S1 and S2). The conventional lesion profile, based on vacuolation score, was similar in BSE- and BASE-infected cattle; however, a more severe involvement of central grey matter (periaqueductal grey) and rostral colliculus but not the vestibular nuclear complex were observed in BASE-inoculated cattle as compared to BSE-challenged animals, which showed severe involvement of the putamen (Figure S1). Additional brain areas, including the olfactory areas, amygdalae, hippocampi and dorsal horns of spinal cords, were severely involved in both groups. Ventral and dorsal roots did not show major pathological changes.

**Figure 3:**
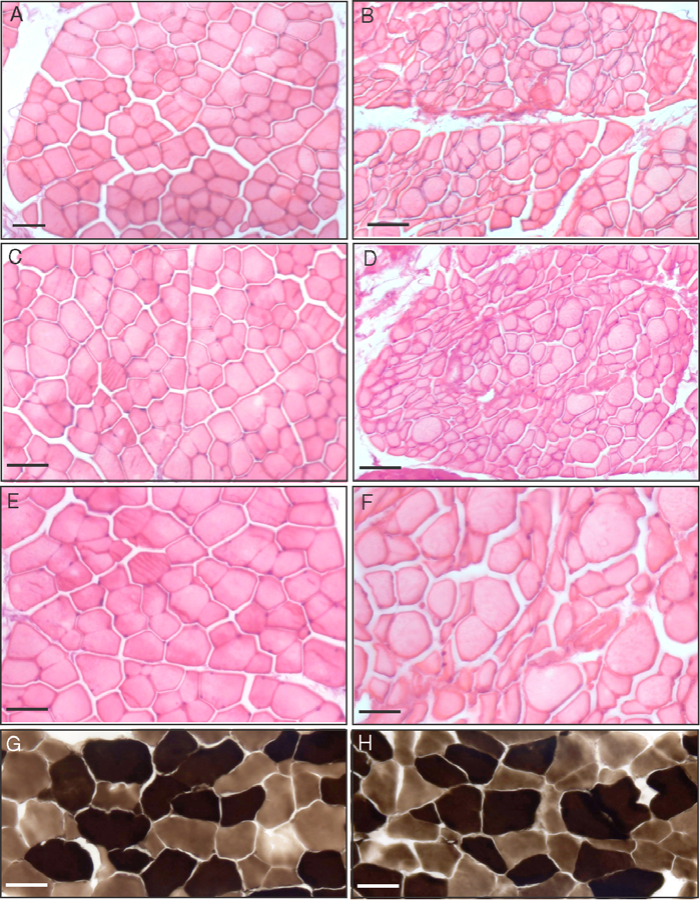
Histology and ATPase staining of muscle sections from *gluteus medius* of BSE and BASE cattle and *major psoas* of BASE cattle. (A) Friesian cattle challenged with BSE and (B) BASE; (C, E) BSE- and (D, F) BASE-infected Alpine brown cattle. As opposed to the normal appearance of muscle tissue in BSE-infected cattle, groups of atrophic muscle fibers are seen in BASE-inoculated cattle. Hematoxylin and eosin staining (A–D scale bars 50 μm; E, F scale bars 20 μm). (G, H) Myosin ATPase staining of major psoas from BASE-infected cattle (code breed g, 995; h, 994) after preincubation at pH 4.6, shows that atrophic muscle fibers are both types I and II (scale bars 50 μm).

Friesian and Alpine brown muscle tissue was normal in BSE-infected cattle (Figure 3A, 3C, 3E and 3G), whereas groups of atrophic muscle fibers were observed in the *gluteus medius* (Figure 3B, 3D and 3F) and, to a decreasing extent, in *major psoas, longissimus dorsi,* and *triceps brachii* of BASE-infected cattle (Figure 3H).

**Figure 4:**
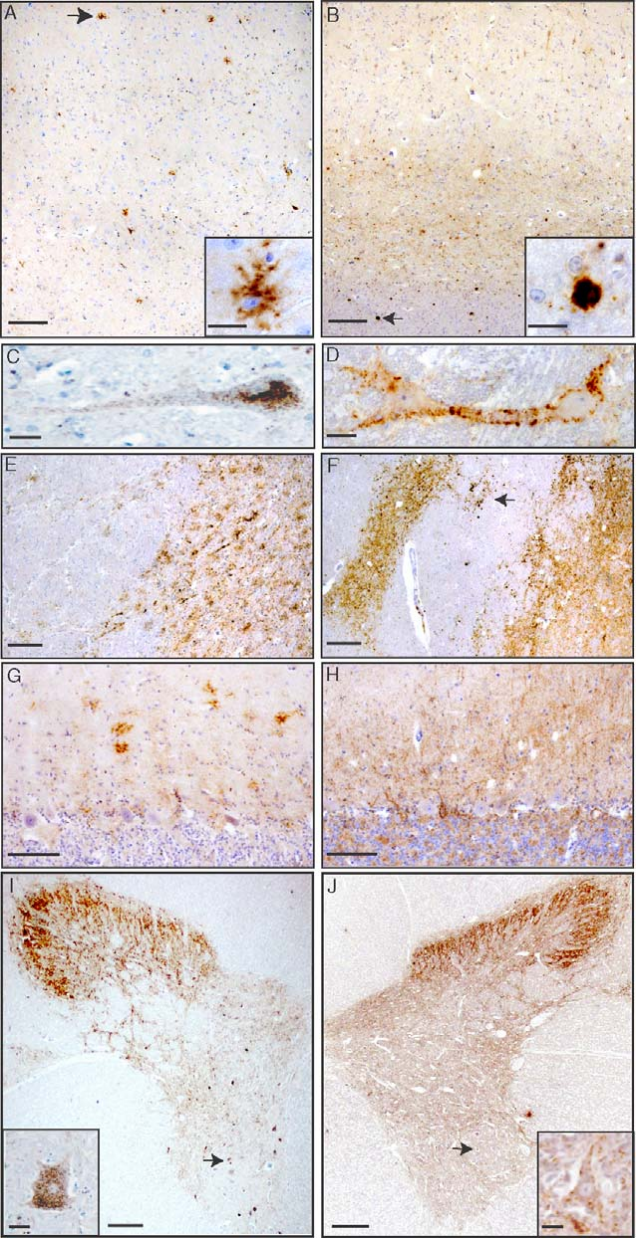
PrP deposition in the brain and spinal cord of BSE and BASE infected cattle. (A, C, E, G, I) BSE infected cattle, showing synaptic-punctuate and stellate patterns in (A) the frontal cortex (scale bar 100 μm), detail of stellate labelling (inset, scale bar 20 μm) and (C) detail of granular staining in the perikaryon of a cortical neuron (scale bars 20 μm); (D, D, F, H, J) BASE cattle showing (B) a diffuse synaptic-punctuate staining and abundant PrP plaques in the deeper cortical layers and in the white matter of frontal cortex (arrow, scale bar 100 μm), detail of a plaque (inset, scale bar 20 μm), and (D) of a cortical neuron (scale bars 20 μm). The different patterns of PrP deposition, in all BSE- and BASE-infected cattle, were also observed in the thalamus (E, F) and in the cerebellum (G, H), with the exception of amyloid plaques which were not observed in the cerebellum (scale bars 100 μm). PrP deposition in spinal cord mainly occurred at the level of Rexed laminae I–III, in both BSE (I) and BASE (J) infected cattle (scale bars 500 μm); motor neurons, indicated by arrows, displayed distinct patterns of PrP deposition in BSE (inset, scale bar 20 μm) and in BASE (inset, scale bar 20 μm) infected cattle (F99, 1∶5000).

In BSE cattle, a synaptic-punctate and “glial-associated” stellate pattern of PrP deposition was observed in different brain areas, including olfactory areas, cerebral cortex, basal ganglia, thalamus, cerebellum, medulla, and spinal cord (Figure 4A and inset, 4E, 4G, 4I and inset). Conversely, in BASE-inoculated cattle, abundant amyloid PrP plaques were observed in subcortical white matter and in deep grey nuclei, as observed in natural BASE cases (Figure 4B and inset, 4F; and Figure S2 and S3). No PrP plaques were seen in the olfactory glomeruli, the cerebellum or the spinal cord (Figure 4I and 4J). Neurons from BSE cattle showed intracellular PrP deposition in contrast to the membrane-associated deposits observed in neuronal cells of BASE cattle (Figure 4C and 4D). These patterns of PrP neuronal staining were also seen in ventral horn neurons of the spinal cord (Figure 4I and J insets) and in the dorsal root ganglion cells (data not shown). No PrP staining was detected in the peripheral nerves and muscles.

## Discussion

In the present work, we demonstrate that BSE and BASE isolates maintain distinct biological properties and induce different disease phenotypes after transmission in their natural host. The similarity of the molecular typing differences between BASE and BSE PrP^TSE^ in Friesian and Alpine Brown cattle also supports the notion that the two conditions are caused by different prion strains.

Cattle inoculated with BASE developed a syndrome characterized by progressive muscle atrophy and behavioural changes. Amyotrophic changes were preceded by fasciculations, findings denoting a lower motor neuron deficit. The absence of anorexia or difficulty in feeding and swallowing suggests that amyotrophy may be caused by motor neuron dysfunction and, therefore, not indicative of a generalized wasting syndrome, such as that observed in chronic wasting disease [15]. Consistent with clinical findings of lower motor neuron involvement, pathological examination of muscle tissues disclosed groups of atrophic fibers more frequently detected in proximal than distal hind limb muscles. However, there was no convincing loss of ventral horn neurons.

Pathogenic mechanisms leading to motor neuron dysfunction remain unknown; however, the role of pathological PrP deposition at the plasma membrane of motor neurons or a loss of PrP function, as observed in experimental models of amyotrophic lateral sclerosis, cannot be ruled out [16].

Clinical signs of motor neuron dysfunction, including stiffness, posterior paresis with “clonic spasms of muscle bundles” [17] and generalized weakness, accompanied by severe lethargy and ataxia, were previously reported in cattle experimentally infected with American strains of sheep scrapie (either at first or at second passage). Cattle inoculated with pre-1975 and post-1990 sources of sheep scrapie from the UK presented similarly with ataxia and weakness and most showed dullness with low head carriage and did not over react to external stimuli [17]–[19]. While the clinical characterization described previously in cattle infected with scrapie is suggestive of upper and lower motor neuron involvement, results obtained in BASE cattle point to lower motor neuron dysfunction or to peripheral neuropathy as the cause of amyotrophic changes.

In contrast to the amyotrophic changes observed in BASE-inoculated cattle, animals inoculated with BSE presented a disorder characterized by apprehension and hypersensitivity to external stimuli, overlapping clinical features described in early accounts of UK BSE [20].

Molecular features of PrP^TSE^ from BASE and BSE donor cattle were preserved with high fidelity in recipient animals. In particular, the conformation of PrP^TSE^, as assessed by the electrophoretic motility of the core fragment, and the glycosylation status were indistinguishable in recipient animals of different breeds compared to the original inocula. These PrP^TSE^ traits were maintained in all cortical and subcortical investigated brain regions.

Taken together, PrP^TSE^ molecular traits and PrP^TSE^ regional distribution showed distinct patterns in the two groups of animals, supporting the notion of two different prion strains, as also suggested by results from experimental transmission to transgenic mice expressing bovine PrP [10],[13].

Differences between BASE- and BSE-inoculated animals were also observed at the neuropathological and immunohistochemical levels. At variance with original BASE cases, where no spongiform changes were observed, as a likely effect of early disease stage, marked vacuolation was seen in BASE-inoculated cattle with a lesion profile divergent from that seen in BSE-treated animals in at least four regions. Indeed, extensive vacuolar pathology was seen in the hindbrain of BASE-inoculated cattle, whereas severe involvement of the putamen was a distinguishing feature in the BSE group.

The divergent biological properties of the two strains were further confirmed by the different patterns of PrP deposition, indistinguishable from those patterns seen in naturally occurring BSE and BASE. Moreover, the distinct neural and microglial cells involved in the two groups and the subcellular sites of PrP accumulation denote a different trafficking and propagation of PrP.

Taken together, intraspecies transmission of BASE and BSE recapitulated the key neuropathological hallmarks observed in these naturally occurring cattle TSEs. This is at variance with the alternate patterns of PrP^TSE^ depositions seen in inoculated Tgbov XV mice, i.e., uni- and multicentric plaques in BSE-challenged animals and diffuse/focal PrP deposition, but not amyloid plaques, in BASE-inoculated mice [10],[13].

We recently showed that in TgBov XV mice challenged with the same inocula used in the present study, BASE-inoculated mice had significantly shorter incubation periods and survival times than BSE-inoculated mice, consistent with results from another laboratory [10],[13]. This effect was not influenced by any species barrier phenomenon and is therefore likely to be strain-dependent. An exception to diverging phenotypic characteristics observed in BSE- and BASE-inoculated cattle was the incubation time observed for BSE-inoculated Alpine brown (but not BSE-treated Friesian animals) which did not significantly differ from times assessed for BASE-inoculated Friesian and Alpine brown cattle.

However, the small number of investigated animals and the individual variability in incubation times dictate caution in the interpretation of these data. In contrast, the breed-associated effect in BASE-inoculated cattle, with significantly shorter incubation periods and survival times in Friesian than in Alpine brown, suggests that disease-modifier genetic loci other than known *PRNP* polymorphisms could be relevant to both of these parameters.

While it is now clear that vCJD originated from human exposure to BSE, it is still uncertain whether emerging cattle TSEs, including BASE, or L-BSE, and H-BSE have infected humans or to which extent they can be potentially dangerous for human and animal health. Recent experimental data show that the BASE strain is efficiently transmitted to Tgbov XV mice and to TgOv mice; in the latter, BASE transmits at first passage with a 100% attack rate, as opposed to cattle BSE that transmits with a low attack rate [21]. Moreover, transmitted BASE shows shorter incubation periods than BSE in *Cynomolgus* monkeys [14]. Paradoxically, while BASE is efficiently transmitted at first passage and with a high attack rate to 129 Met/Met Tg humanized mice [22], human transgenic lines of all genotypes at codon 129 are resistant to BSE transmission [22],[23]. Taken together, these data might suggest that the BASE agent could transmit to humans more efficiently than the BSE agent.

## Materials and Methods

### Animal care

All procedures involving animals and their care were conducted in conformity with national and international laws and policies (EEC Council Directive 86/609, OJL358, 1, 12 December 1987; Italian Legislative Decree 116/92, Gazzetta Ufficiale della Repubblica Italiana 10, 18 February 1992; and *Guide for the Care and Use of Laboratory Animals,* U.S. National Research Council, 1996), and the study was approved by the authors' Institutional Review board.

### Bovine PrP Gene Determination

*PRNP* ORF amplification, sequencing and determination of the octapeptide repeat copy number was performed as previously described [12]. Polymorphisms of the 12-bp indel, located within intron 1, and 23-bp indel, located in the promoter region, were determined as previously reported [24]. Primer pairs 5′-CCTGTTGAGCGTGCTCGT/5′-ACCTGCGGCTCCTCTACC-3′ and 5′-GAAGTCACGTGAAGGCACT-3′/5′-CAAAGAGTTGGACAGGCACA-3′ were used to amplify the 12-bp indel (202 bp/214 bp) and 23-bp indel (167 bp/190 bp), respectively, as described above. PCR was performed as 30 cycles of 30 sec at 94°C, 30 sec at 55°C and 45 sec at 72°C. High resolution agarose (3.5%) gel electrophoresis was used to visualise the allelic PCR products whose specificity and length was also confirmed by direct sequencing with the same primer used for the PCR described above.

### Inocula

10% brain homogenates from the thalamus of a BSE-affected Friesian (code #128204) and a BASE-affected Piemontese (code #1088) were prepared in phosphate-buffered saline. These cattle carried the same PrP genotype with six octapeptide repeats, and were extensively studied in our previous work [12]. BSE and BASE inocula were prepared to obtain a comparable amount of PrP^TSE^ as assessed by Western blot analysis with the 6H4 anti-PrP monoclonal antibody (Prionics).

### Inoculation of cattle

Eight Friesian and six Alpine brown cattle (4 months old) were purchased from Italian herds in which no cases of BSE had ever been recorded. All calves were free of neurological signs. Prior to inoculation, animals remained in the new environment for one month for adaptation. Inoculation was carried out using a semi-stereotaxic technique in surgical aseptic conditions. Calves were anesthetized with xylazine (50 μg/kg), a midline incision was made at the junction of the parietal and frontal bones, and a 1-mm hole was drilled through the calvarium. The inoculum was injected into the frontal lobe via a 22-gauge 9-cm-long disposable needle while the needle was withdrawn. Two groups of animals, each comprising three Friesians and three Alpine brown cattle, were inoculated with 1 milliliter of 10% brain homogenate from BSE-and BASE-affected animals, respectively. Conversely, two Friesians cattle were challenged with 1 milliliter of phosphate buffered saline and used as controls. To avoid potential cross-contamination, BSE and BASE transmission experiments were performed on different days and the facility was decontaminated with 10% sodium hypochlorite solution after each inoculation.

### Clinical assessment

Clinical evaluations were comprised of a bi-weekly observation by the veterinarian and two daily observations by animal husbandry staff who reported any observed motor and/or behavioural changes. For assessment of the gait cows were walked along the corridor outside the pen. The cattle were filmed nightly with closed circuit television monitoring to record signs of disease that may not have been observed during the day. Once a month, a veterinarian trained in neurology examined each cattle by conventional neurological scale evaluations [25],[26]. Animals were considered symptomatic when they showed two of the following neurological signs observed in two separate consecutive examinations: abnormal behaviour, abnormal posture, aberrant reactions, or hyperreactivity to sensitive stimuli, light and sound.

### Postmortem investigations

Cattle at the terminal stage were euthanized with pentobarbital administered intravenously. Peripheral organs, including cervical and mesenteric lymph nodes, spleen, thymus, liver, lung, peripheral nerves and forelimb and hind limb muscles (*m. triceps brachii, m. longissimus dorsi, m. gluteus medius and m.major psoas*), were sampled and each sample was divided equally; one portion was fixed in 4% buffered formaldehyde for H&E stain and PrP immunohistochemistry, and the other was frozen. Serial 10-μm-thick muscle cryosections were stained with H&E and adenosine triphosphatase (ATPase), after pre-incubation at pH 4.3, 4.6 and 10.4. Nervous tissue was removed in a separate area to avoid cross-contamination. The fixed half of the brain sample was used for neuropathological examination, while the frozen brain sample was stored at −80°C for biochemical analyses. The spinal cord was sampled at cervical, thoracic, lumbar and sacral levels and sections were fixed in 10% buffered formaldehyde. The remaining tissue was frozen at −80°C for further studies.

### Immunoblot analysis

From each neural tissue sample, including optic nerve, olfactory bulb, frontal cortex, occipital cortex, hippocampus, nucleus caudatus, putamen, globus pallidus, thalamus, cerebellum, obex, and cervical, thoracic, lumbar and sacral spinal cord, 100 mg of wet tissue was homogenized in 9 volumes of lysis buffer (100 mM sodium chloride/10 mM EDTA/0.5% Nonidet P-40/0.5% sodium deoxycholate/10 mM Tris, pH 7.4) and digested with 50 μg/ml of proteinase K (Boehringer Mannheim) for 1 h at 37 °C. Digestion was blocked by the addition of phenylmethylsulfonyl fluoride at 2 mM. For deglycosylation, proteinase K-digested samples were deglycosylated with recombinant peptide *N*-glycosidase F (PNGase F) according to manufacturer's instructions (Boehringer Mannheim). Samples, equivalent to 400 μg of wet tissue, were resolved on 13% polyacrylamide gels and then transferred onto PVDF membrane (Immobilon P; Millipore, Bedford MA) for 2 hours at 60V. Membranes were blocked with 1% non-fat dry milk in TBST (10 mM Tris/150 mM sodium chloride/0.1% Tween-20, pH 7.5) for 1 hour at 37°C and incubated overnight at 4°C with anti-PrP monoclonal antibody 6H4 (Prionics) diluted to 1/5,000. Blots were developed using the Amersham enhanced chemiluminescence (ECL) system, as described by the supplier and visualized on an autoradiographic film. Films were scanned by using a densitometer (GS-710, Biorad), calculating the relative amounts of PrP^Sc^ in a semiquantitative manner.

To enhance PrP^TSE^ detection, extraneural tissues, including cervical and mesenteric lymph nodes, spleen, thymus, liver, lung, peripheral nerves and forelimb and hind limb muscles, that were negative on a standard immunoblot were subjected to phosphotungstic acid (PTA) precipitation and analyzed by Western blot, as previously described [27]. Briefly, 100 mg of wet tissue were homogenized in nine volumes of 2% sarkosyl in phosphate-buffered saline, pH 7.4. Cellular debris were removed by centrifugation at 1,000 rpm for 2 minutes and samples were incubated for 30 minutes at 37°C with constant agitation in phosphate-buffered saline containing 50 units/mL Benzonase and 1 mmol/L magnesium chloride. Subsequently, samples were adjusted to 0.3% sodium phosphotungstic acid, incubated at 37°C for 30 minutes and centrifuged at 14,000 rpm for 30 minutes. The supernatant was saved, and the pellet dissolved in 20 μl phosphate-buffered saline, pH 7.4, containing 0.1% sarkosyl. The supernatant and the pellet were adjusted to a final concentration of 20 μg of proteinase K per milliliter and incubated at 37 °C for 30 minutes.

### Neuropathology and PrP immunohistochemistry

Three-mm thick samples were embedded in paraffin after decontamination with 98% formic acid for 1 hour. The paraffin-embedded blocks selected for the study included coronal sections at the level of the olfactory bulb, the frontal, parietal and occipital cortices, the pyriform lobus, hippocampus, striatum, thalamus, brainstem, sagittal sections through the cerebellum and spinal cord at cervical, thoracic, lumbar and sacral levels. Histological sections were deparaffinized, rehydrated, and stained with hematoxylin and eosin. Additional sections were stained with thioflavin S. The distribution of spongiosis, was determined by using a conventional lesion profile, which allows to characterize strain tropism and to compare the present results with those of previous studies on field and experimental BSE [28]. The definition of each score was performed by three independent observers blinded to the animal identification, as follows: 0 no vacuolation, 1 a few vacuoles (minimum 3 per field×10 objective), 2 several vacuoles evenly distributed, 3 moderate numbers or many vacuoles evenly distributed, and 4 numerous vacuoles some of which coalescing, as previously described [28].

For immunohistochemical study, sections obtained form nervous and extraneural tissues, were rehydrated and treated with 98% formic acid for 20 min at room temperature, followed by hydrated autoclaving in distilled water at 121°C for 30 min. After rinsing, sections were incubated overnight at 4°C with anti-PrP monoclonal antibody F99/97.6.1 (VMRD, inc.; diluted to 1/1,000), recognizing a conserved epitope (QYQRES) on the cattle PrP. Subsequent antibody detection was carried out using a biotinylated goat anti-mouse secondary antibody diluted to 1/200 for 20 min (Vector Laboratories, Burlingame, CA) at room temperature, followed by the avidin-biotin-peroxidase complex (Vectastain ABC kit; Vector Laboratories) according to manufacturer's protocol. Immunoreactivity was visualized using 3,3′-diaminobenzidine as chromogen.

## Supporting Information

Figure S1 Lesion profiles and pathological changes in BSE and BASE challenged cattle. 1. Nucleus of the solitary tract. 2. Nucleus of the spinal tract of the trigeminal nerve. 3. Hypoglossal nucleus. 4. Vestibular nuclear complex. 5. Cochlear nucleus. 6. Cerebellar vermis. 7. Central grey matter. 8. Rostral colliculus. 9. Medial geniculate nucleus. 10. Hypothalamus. 11. Nucleus dorsomedialis thalami. 12. Nucleus ventralis lateralis thalami. 13. Frontal cortex. 14. Septal nuclei. 15. Caudate. 16. Putamen. 17. Claustrum; (A,B and C,D) represent the histopathological changes in areas 7 and 16, respectively, included in the lesion profiles in BSE (A and C) and BASE (B and D) infected cattle; (E and F) hippocampus, which is not included among the brain areas of lesion profile, shows consistent spongiform changes in both BSE (E) and BASE (F) infected cattle (scale bars 100 μm).
(2.93 MB TIF)

Figure S2 Immunocytochemistry for GFAP in BSE- (A, C, E) and BASE-infected cattle (B, D, F). Central grey matter (7) and frontal cortex (13) show a higher degree of gliosis in BASE, whereas the putamen is more affected in BSE-infected cattle (17; scale bars 75 μm).
(6.18 MB TIF)

Figure S3 Tinctorial properties of amyloid plaques in BASE. Fluorescent Kuru-plaques in the frontal cortex of a Friesian (code: # 259) and Alpine brown (code: # 994) BASE-infected cattle (A, B; thioflavin S method).
(0.49 MB EPS)

Video S1 Clinical phenotype of BSE-infected cattle.
(2.24 MB MOV)

Video S2 Clinical phenotype of BASE-infected cattle.
(3.39 MB MOV)
