# Supplementary figures and images for: Intraspecies Transmission of BASE Induces Clinical Dullness and Amyotrophic Changes
Source: PLoS Pathog. 2008 May 23;4(5):e1000075. doi: 10.1371/journal.ppat.1000075 (PMC2374911; doi:10.1371/journal.ppat.1000075)

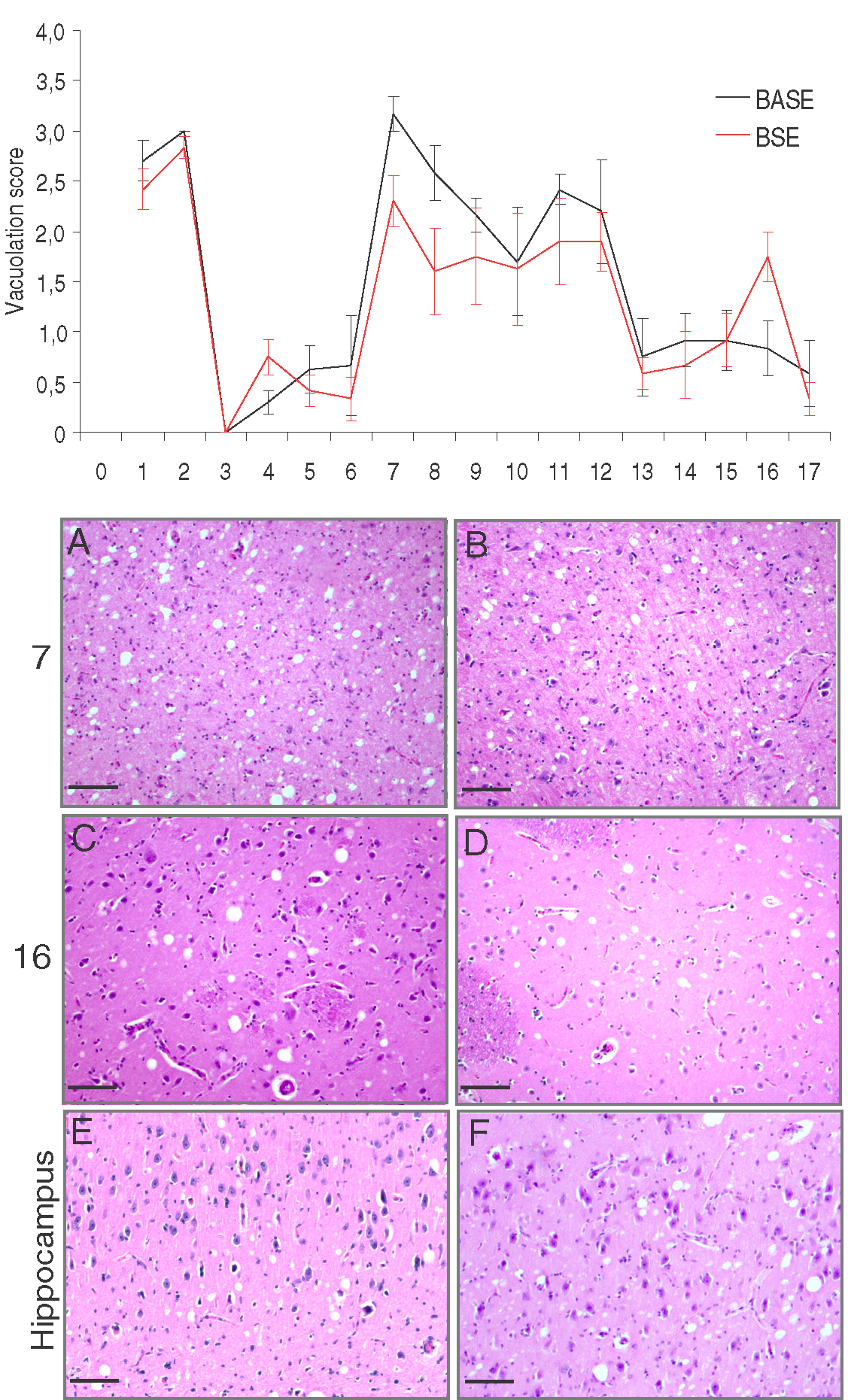

Supplement: Figure S1 — Lesion profiles and pathological changes in BSE and BASE challenged cattle. 1. Nucleus of the solitary tract. 2. Nucleus of the spinal tract of the trigeminal nerve. 3. Hypoglossal nucleus. 4. Vestibular nuclear complex. 5. Cochlear nucleus. 6. Cerebellar vermis. 7. Central grey matter. 8. Rostral colliculus. 9. Medial geniculate nucleus. 10. Hypothalamus. 11. Nucleus dorsomedialis thalami. 12. Nucleus ventralis lateralis thalami. 13. Frontal cortex. 14. Septal nuclei. 15. Caudate. 16. Putamen. 17. Claustrum; (A,B and C,D) represent the histopathological changes in areas 7 and 16, respectively, included in the lesion profiles in BSE (A and C) and BASE (B and D) infected cattle; (E and F) hippocampus, which is not included among the brain areas of lesion profile, shows consistent spongiform changes in both BSE (E) and BASE (F) infected cattle (scale bars 100 μm). (2.93 MB TIF) [file ppat.1000075.s001.tif]

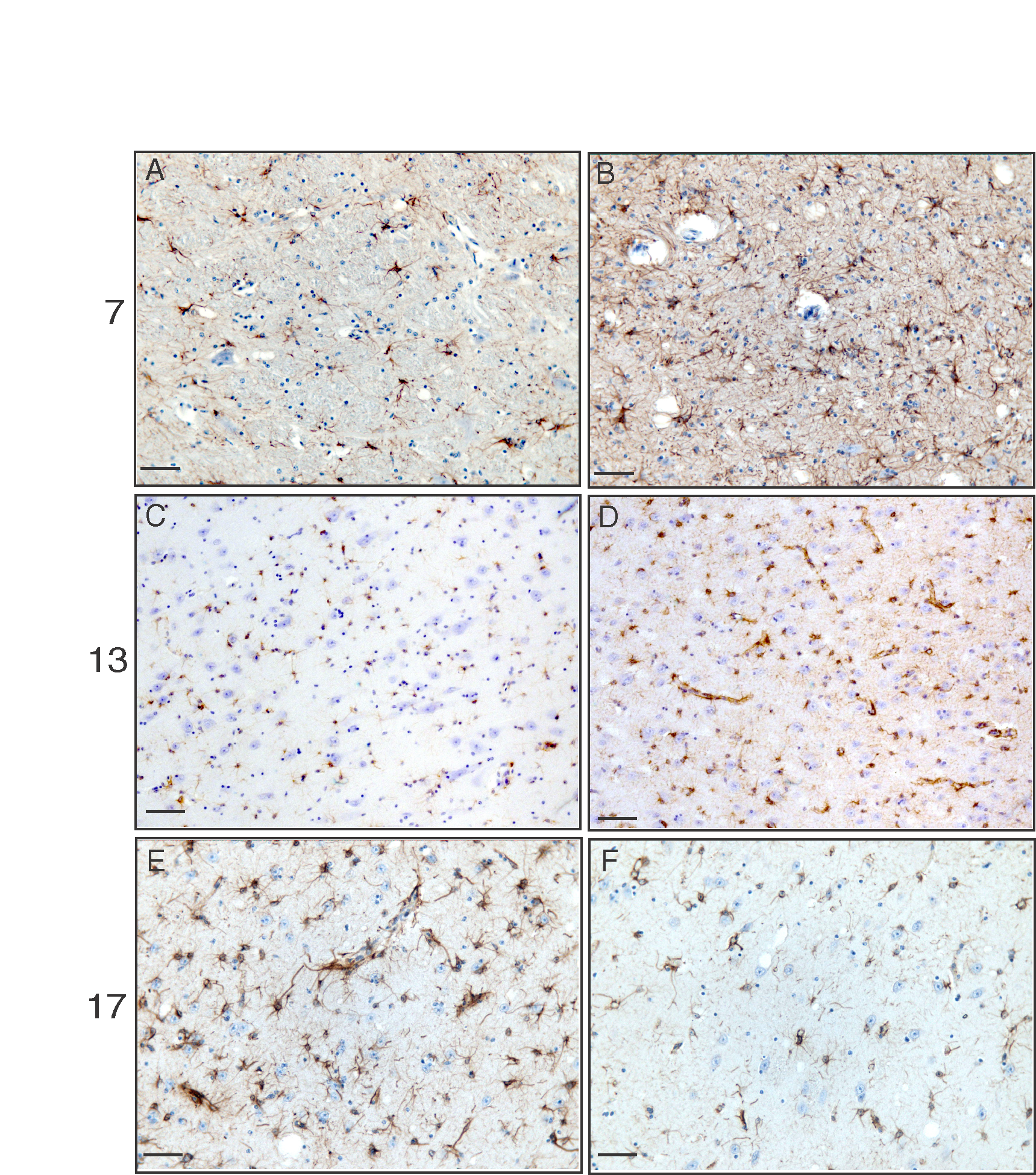

Supplement: Figure S2 — Immunocytochemistry for GFAP in BSE- (A, C, E) and BASE-infected cattle (B, D, F). Central grey matter (7) and frontal cortex (13) show a higher degree of gliosis in BASE, whereas the putamen is more affected in BSE-infected cattle (17; scale bars 75 μm). (6.18 MB TIF) [file ppat.1000075.s002.tif]
